# The functions and mechanisms of long non-coding RNA in colorectal cancer

**DOI:** 10.3389/fonc.2024.1419972

**Published:** 2024-07-04

**Authors:** Yuning Lin, Wenzhen Zhao, Zhenyi Lv, Hongyan Xie, Ying Li, Zhongying Zhang

**Affiliations:** ^1^ Medical Laboratory, Xiamen Humanity Hospital, Fujian Medical University, Xiamen, China; ^2^ Ultrasonography Department, Women and Children’s Hospital, School of Medicine, Xiamen University, Xiamen, China

**Keywords:** lncRNA, colorectal cancer, biomarker, functions, mechanisms

## Abstract

CRC poses a significant challenge in the global health domain, with a high number of deaths attributed to this disease annually. If CRC is detected only in its advanced stages, the difficulty of treatment increases significantly. Therefore, biomarkers for the early detection of CRC play a crucial role in improving patient outcomes and increasing survival rates. The development of a reliable biomarker for early detection of CRC is particularly important for timely diagnosis and treatment. However, current methods for CRC detection, such as endoscopic examination, blood, and stool tests, have certain limitations and often only detect cases in the late stages. To overcome these constraints, researchers have turned their attention to molecular biomarkers, which are considered a promising approach to improving CRC detection. Non-invasive methods using biomarkers such as mRNA, circulating cell-free DNA, microRNA, LncRNA, and proteins can provide more reliable diagnostic information. These biomarkers can be found in blood, tissue, stool, and volatile organic compounds. Identifying molecular biomarkers with high sensitivity and specificity for the early and safe, economic, and easily measurable detection of CRC remains a significant challenge for researchers.

## Introduction

CRC is a common type of cancer, ranking as the third most prevalent malignant tumor globally and the second leading cause of cancer-related deaths (1, 2). The survival rate of CRC patients is significantly influenced by the stage at which the tumor is detected, with a general 5-year overall survival rate of about 65% (3). Most cases of CRC are diagnosed at an advanced stage due to the lack of unique, early-stage disease-specific symptoms and limitations in early diagnosis methods. The most critical portion of cancer-related deaths is caused by metastatic cancer, with the liver being the most common site of metastasis in CRC. More than half of the patients in stage III, and a quarter in stages II and III, appear to have local recurrence or metastasis (4). CRC is a unique cancer that can be prevented and cured through early identification and removal of high-risk adenomas (5). Thus, implementing early detection screening programs is crucial for reducing the incidence and mortality of the disease. Early detection increases the likelihood of successful treatment and improves patient health outcomes (6). Colonoscopy is a widely accepted and effective screening method for early detection of CRC, though it carries certain risks. Patients may experience bleeding during sampling or polyp removal, and other complications may occur (7). In recent years, advanced molecular techniques have played a significant role in the early diagnosis and treatment of various cancers, including CRC, helping to reveal some of the genetic mechanisms that lead to CRC (8). Therefore, clarifying the molecular mechanisms of colon cancer occurrence is crucial. In recent years, non-coding RNAs (ncRNAs) have been proven to participate in the onset and progression of colon cancer (9, 10). It is well-known that ncRNAs, mostly not translated into proteins, also play significant roles in various cellular and physiological processes (11). LncRNAs are longer than 200 nucleotides, participate in various biological processes including cell proliferation, differentiation, development, apoptosis, and metastasis, often acting as competitive endogenous RNAs (ceRNAs) to regulate the expression of specific miRNAs, then targeting molecules downstream of these miRNAs (12). In fact, lncRNAs can interact with RNA, DNA, and proteins to form RNA-RNA, RNA-DNA, and RNA-protein complexes, regulating gene expression through various mechanisms, including transcriptional regulation, mRNA stability, and translation (13, 14). Numerous studies suggest that lncRNAs may play key roles in the biological processes of cancer, such as apoptosis, cell proliferation, cell invasion, and metastasis (15–17). In this article, we will summarize the functions and mechanisms of lncRNAs in the occurrence and malignant progression of human CRC.

## The history of lncRNAs

LncRNAs is a class of RNA molecules longer than 200 nucleotides, first discovered in the 1970s. Initially, scientists primarily focused on mRNA, which encodes proteins, while non-coding RNAs were considered “noise” or “by-products.” However, with technological advancements and deeper research, it gradually became apparent that non-coding RNAs play crucial roles in gene regulation, epigenetics, and disease occurrence. The research journey of LncRNA can be traced back to a series of groundbreaking studies in the late 20th and early 21st centuries. In 2002, researchers first discovered a LncRNA associated with gene silencing on the X chromosome (18). Subsequently, Guttman et al. discovered the LncRNA-HOTAIR, which plays a significant role in gene locus regulation (11). In 2009, Rinn et al. identified an lncRNA (HOTTIP) located in the HOX gene cluster and found its crucial involvement in gene locus regulation (19). It has also been reported that LncRNA plays crucial roles in embryonic development (20). Studies have also indicated the role of lncRNA in tumor initiation and progression, sparking a research frenzy into the role of lncRNA in cancer (12, 21, 22).

## Classification of lncRNAs

Based on the genomic database [Ensembl Release 96 (April 2019)], human lncRNAs are classified into several categories, including 3’ overlapping ncRNA, antisense LncRNA, long interspersed ncRNA, retained intron, sense intronic, sense overlapping, and macro lncRNAs.Intronic lncRNA is located within the intron regions of protein-coding genes, transcribed from the introns of these genes, but does not itself participate in encoding proteins (23). Antisense lncRNA overlaps with the antisense strand of coding genes and may affect gene expression by forming double-stranded RNA structures with coding regions through base complementary pairing (24, 25). Intergenic lncRNA is located in the region between two coding genes and may play a role in the regulation of genes in its region (11), sense lncRNAs are overlapped with the sense strand of protein coding genes containing exons (26). Messenger lncRNA can act as a regulatory factor, involved in regulating the expression of specific genes (20). Structural lncRNA may play an important role in the physical structure within cells or the chromosomal architecture within the nucleus (27).

## lncRNAs localization and related research techniques

lncRNAs can be located in the cytoplasm (28), nucleus (29), nucleolus (30)s, and other subcellular regions and vesicles (such as nucleoli and exosomes), and their localization is related to their molecular functions (28, 31). Certain sequence motifs in their primary sequences are related to subcellular localization (32). Exploring the localization of lncRNAs is important for understanding their roles in gene regulation, disease development, and cell functions. Techniques for studying the localization of lncRNAs include *in situ* hybridization (33), RNA immunoprecipitation (34), RNA-seq (35), single-cell RNA sequencing (36), FISH-Flow (37), etc.

## The conservation of lncRNAs

Although lncRNAs are crucial in function, most lncRNA sequences exhibit low conservation across different species, meaning the same lncRNA may be difficult to identify in different species through sequence similarity. Low conservation is considered to reflect the diversity and specificity of lncRNA functions, as well as their rapid changes during evolution (38). Despite low sequence conservation, some lncRNAs exhibit a degree of structural and functional conservation across different species. These lncRNAs may maintain similar three-dimensional structures or play roles in the same pathways of gene expression regulation across species (27, 39). Moreover, many lncRNAs exhibit strong species specificity, existing in certain species while absent in others. This species specificity suggests that lncRNAs may play specialized roles in the development and adaptation processes of specific species (38, 40). The conservation level of lncRNA promoters is comparable to that of protein-coding genes (41, 42).

## LncRNAs as diagnostic biomarkers for CRC in blood

Ease of acquisition and detectability are preferred criteria for diagnostic biomarkers. For potential early-stage CRC patients, undergoing a colonoscopy to obtain tissue samples might be strongly resisted by patients. Therefore, biomarkers that can be detected in blood or other body fluids are ideal for broader clinical use. Over the past decade, numerous studies have demonstrated that lncRNAs are stable in the bloodstream and hold diagnostic potential. This makes lncRNAs promising candidates for non-invasive diagnostic tests in CRC (43–46). LncRNAs are present in different body fluids such as blood and urine because they can cross cellular membranes. This property allows for their detection in non-invasive diagnostic tests (47). LncRNAs in body fluids directly represent the expression levels of certain genes and can differentiate between cancer patients and healthy individuals (48). Additionally, a key characteristic of circulating lncRNAs is their ability to resist degradation by RNase enzymes (49, 50). Apoptotic bodies, microvesicles, and exosomes are vesicles encapsulated by a phospholipid bilayer that contain DNA, RNA, lipids, proteins, polysaccharides, and metabolites. These vesicles are released into the human circulatory system for dissemination, facilitating the transfer of materials between cells (51–53). Quantitative reverse transcription PCR (qRT-PCR) is frequently employed to detect circulating lncRNAs because of its notable sensitivity and specificity (54). CCAT1 and HOTAIR are the first lncRNA markers reported to show significantly higher levels in the plasma of CRC patients compared to healthy individuals (55). Numerous other circulating lncRNAs have also been identified as potential biomarkers for detecting CRC (**Table 1**).

**Table 1:** Studies on LncRNAs as diagnostic biomarkers for CRC.

Sample Type	Biomarker(S)	Diagnostic (AUC)	Study sample size (Cancer vs Control)	Ref
serum	CCDC144NL-AS1	0.994	60 vs 30	(56)
serum	TERC	0.982	70 vs 35	(57)
serum	NNT-AS1	0.964	60 vs 28	(58)
Serum	circRHBDD1	0.76	24 vs 24	(59)
serum	ZFAS1	0.95	60 vs 28	(60)
Plasma	LncGMDS-AS1	0.721	97 vs 91	(61)
Plasma	CACClnc	0.846	22 vs 22	(62)
Serum	EGFR-AS1	0.938	128 vs 64	(63)
Plasma	ARST	0.934	60 vs 60	(64)
Plasma Tissue	ASB16-AS1 AFAP1-AS1	Tissue.LncRNA.ASB16-AS1:0.996 Plasma.LncRNA.ASB16-AS1:0.974 Tissue.LncRNA.AFAP1-AS1 :0.984 Plasma.LncRNA.AFAP1-AS1:0.965	47 vs 50	(65)
serum	LINC01836	0.809	137 vs 138	(66)
serum	LNCAROD SNHG20 LINC00534 TSPOAP-AS1	LNCAROD:0.74 SNHG20:0.73 LINC00534:0.73 TSPOAP-AS1:0.63	45 vs 45	(67)

## The role and regulatory axes of LncRNAs in CRC

As research progresses, it has been discovered that lncRNAs play a crucial role in the onset and development of CRC (68). LncRNAs play multifaceted roles in CRC, influencing various biological processes including cell cycle control, cell proliferation, epithelial-mesenchymal transition (EMT), migration, invasion, drug resistance, apoptosis, and cellular stemness. This section will elaborate on the specific roles of lncRNAs within CRC, highlighting how they contribute to both the development and progression of the disease. We will also focus on the signaling pathways associated with lncRNAs, enhancing our understanding of their mechanistic impact on the pathophysiology of CRC (**Table 2**).

**Table 2:** The functions of lncRNAs and their related regulatory axes.

LncRNA	Sample	Exp	Functions	Related regulatory axes	Ref
AC092894.1	cell line	↓	Inhibit drug resistance	AC092894.1/USP3/AR/RASGRP3	(69)
AGAP2-AS1	cell line	↑	Promote growth,Promote migration,Promote invasion,Promote EMT	AGAP2-AS1/miR-182-5p/CFL1	(70)
AK077216	tissue	↓	Inhibit migration,Inhibit invasion	AK077216/miR-34a	(71)
AK093407	tissue,cell line	↑	Promote proliferation,Inhibit apoptosis,Promote cell cycle	AK093407/p-STAT3	(72)
ASB16-AS	cell line	↑	Promote proliferation,Promote migration,Promote invasion,Promote stemness,Inhibit apoptosis	ASB16-AS1/miR-185-5p/TEAD1	(73)
ATB	tissue,cell line	↑	Promote invasion, induces EMT	E-cadherin	(74)
BBOX1-AS1	cell line	↑	Promote proliferation,Promote migration,Promote invasion,Inhibit apoptosis	BBOX1-AS1/miR-361-3p/SH2B1	(75)
BCAR4	tissue	↑	Inhibit apoptosis,Promote drug resistance	BCAR4/miR-483-3p/RAB5C	(76)
BVES-AS1	cell line	↑	Enhance cell vitality,Promote migration,Promote invasion	BVES-AS1-201-50aa/Src/mTOR	(77)
CASC2	tissue,cell line	↓	Inhibit proliferation,Inhibit migration,Inhibit invasion	CASC2/miR-18a-5p/BTG3	(78)
CASC9	tissue,cell line	↑	Promote proliferation,Inhibit apoptosis	CASC9/miR-576-5p/AKT3	(79)
	tissue,cell line	↑	Promote proliferation,Promote invasion	CASC9/miR-542-3p/integrin-linked kinase	(80)
CBR3-AS1	tissue	↓	Inhibit migration,Inhibit invasion	CBR3-AS1/miR-29a	(81)
CCAT1	cell line	↑	Promote drug resistance	CCAT1/DNMT1/SOCS3	(82)
	tissue	↑	Promote proliferation,Promote invasion	c-Myc/CCAT1	(83)
	tissue,cell line	↑	Promote drug resistance	/	(84)
	tissue	↑	Promotes proliferation,invasion, drug resistance	/	(85)
CCAT2	tissue	↑	Promote growth,Promote migration	CCAT2/miR-17-5p/TCF7L2	(86)
CCL14-AS	tissue	↓	Inhibit migration,Inhibit invasion	CCL14-AS/MEP1A	(87)
CERS6-AS1	tissue,cell line	↑	Enhance vitality, Promote proliferation, Promote migration, Promote invasion, Promote EMT, Promote stemness	CERS6-AS1/miR-15b-5p/SPTBN2	(88)
CRNDE	cell line	↑	Promote proliferation,Inhibit invasion, Promote drug resistance	CRNDE/Akt/mTORC1	(89)
CYP1B1-AS1	tissue	↓	Inhibit proliferation,Inhibit migration,Inhibit invasion	CYP1B1-AS1/NOP58/SNAIL	(90)
CYTOR	cell line	↑	Promotes migration,invasion, EMT	CYTOR/β-catenin/TCFcomplex	(91)
DANCR	tissue,cell line	↑	Promote proliferation,Promote migration, Promote invasion,Promote cell cycle	DANCR/miR-185-5p/HMGA2	(92)
DICER1-AS1	tissue	↑	Promote proliferation,Promote migration,Promote invasion	DICER1-AS1/miR-650/MAPK/ERK	(93)
Duxap8	tissue,cell line	↑	Promote proliferation,Inhibit apoptosis	Duxap8/miR-519b-3p/ZEB1	(94)
	tissue,cell line	↓	Inhibit vitality,Inhibit growth,Inhibit drug resistance	ELFN1-AS1/EZH2	(95)
	tissue、cell line	↑	Promote immune surveillance escape,Inhibit NK cell cytotoxicity	ELFN1-AS/GDF15/JNK/NKG2D/GZMB	(96)
EPB41L4A-AS1	tissue	↑	Promote proliferation,Promote migration, Promote invasion,Promote EMT	EPB41L4A-AS1/Rho/Rh	(97)
EVADR	cell line	↑	Promote cell cycle,Promote migration	EVADR/miR-7 or EVADR/miR-29b	(98)
	tissue,cell line	↑	Promote migration	EVADR/YBX	(99)
FAM201A	tissue,cell line	↑	Promote growth,Promote stemness,Promote drug resistance	FAM201A/miR-3163/MACC1	(100)
FAM230B	tissue	↑	Inhibit proliferation,Enhance vitality,Promote colony formation	FAM230B/miR-1182	(101)
FAM83H-AS1	tissue	↑	Promotes tumorigenesis	SMAD1/5/9,TGF-βsignaling	(102)
FER1L4	tissue	↓	Inhibit migration,Inhibit invasion	FER1L4/miR-1273g-3p	(103)
	tissue	↓	Inhibit proliferation,Inhibit migration,Inhibit invasion	FER1L4/miR-106a-5p	(104)
FEZF1-AS1	tissue	↑	Promote proliferation,Promote migration,Promote invasion	FEZF1-AS1/miR-92b-3p/ZIC5	(105)
	tissue,cell line	↑	Promote proliferation,Promote migration, Promote invasion,Promote tumorigenesis	FEZF1-AS1/miR-632/FAM83A	(106)
FGD5-AS1	cell line	↑	Promote drug resistance	FGD5-AS1/miR-330-3p/Hexokinase 2	(107)
FOXP4-AS1	tissue,cell line	↑	Promote proliferation,Promote migration,Promote invasion,Inhibit apoptosis	FOXP4-AS1/miR-423-5p/NACC1	(108)
GAS5	tissue,cell line	↓	Inhibit migration, Inhibit vitality, Promote apoptosis	GAS5/miR-21/LIFR	(109)
	cell line	↓	Inhibit proliferation,Inhibit migration,Inhibit invasion,Inhibit cell cycle	GAS5的/miR-21/PTEN/Akt	(110)
GAS6-AS1	cell line	↑	Promote proliferation,Promote migration,Promote invasion,Promote EMT	GAS6-AS1/TRIM14	(111)
GSEC	cell line	↑	Promotes migration	DHX36	(112)
H19	tissue	↑	Promote drug resistance	H19/miR-675-5p/VDR	(113)
	cell line	↑	Promote stemness	Sohlh2/LncRNA-H19/miR-141/β-catenin	(114)
HAND2	tissue	↓	Inhibit proliferation,Inhibit migration, Promote apoptosis,Inhibit xenotransplantation	HAND2-AS1/miR-3118/LEPR	(115)
HCG1	cell line	↑	Promote proliferation,Promote migration,Promote invasion,Inhibit apoptosis	HCG1/miR-26b-5p/ARPP19	(116)
HCG11	tissue,cell line	↑	Promote proliferation,Promote migration, Promote invasion,Promote drug resistance	HCG11/miR-144-3p/PDK4	(117)
HNF1A-AS1	tissue	↑	Enhance vitality,Promote migration,Promote invasion,Promote xenotransplantation	HNF1A-AS1/miR-34a/SIRT1/p53	(118)
HOTAIR	cell line	↑	Enhance vitality,Promote proliferation	HOTAIR/miR-1277-5p/ZEB1	(119)
	tissue	↑	Promote stemness	HOTAIR/miR-211-5p/FLT-1	(120)
	tissue	↑	Promote tumorigenesis,Promote migration		(121)
	tissue	↑	Promote EMT	Vimentin,Matrix Metallopeptidase 9 (MMP9),E-cadherin	(122)
	tissue	↑	Promote migration	/	(123)
	tissue,cell line	↑	Promote growth,Promote migration,Promote EMT	HOTAIR/SNAIL/HNF4α	(124)
HOXB-AS4	cell line	↑	Promote proliferation,Promote migration, Inhibit apoptosis	HOXB-AS4/miR-140-5p/HDAC7	(125)
HOXC-AS3	tissue	↓	Inhibit migration,Inhibit invasion	HOXC-AS3/miR-1269/TGF-β2	(126)
IGFL2-AS1	tissue,cell line	↑	Promote proliferation	IGFL2-AS1/miR-433-3p/PAK4	(127)
	tissue,cell line	↑	Promote proliferation,Promote migration,Promote invasion	IGFL2-AS1/HIF-1α/CA9	(128)
KCNQ1OT1	tissue	↓	Inhibit drug resistance	KCNQ1OT1/miR-34a/ATG4B	(129)
LBX2-AS1	tissue	↑	Promote growth,Promote proliferation, Promote migration,Inhibit invasion	LBX2-AS1/miR-627-5p/RAC1/PI3K/AKT	(130)
LEF1-AS1	tissue,cell line	↑	Promote proliferation,Promote migration,Promote invasion	LEF1-AS1/LEF1/FUT8	(131)
LINC00174	tissue,cell line	↑	Promote proliferation,Promote migration,Promote invasion,Inhibit apoptosis	LINC00174/miR-2467-3p/USP21	(132)
LINC00261	cell line	↓	Promote apoptosis,Inhibit vitality,Inhibit migration,Inhibit invasion,Inhibit drug resistance	LINC00261/β-catenin/Wnt-pathway	(133)
LINC00473	tissue,cell line	↑	Promote EMT,Promote proliferation, Promote cell cycle,Promote invasion	LINC00473/miR-195	(134)
LINC00485	tissue,cell line	↓	Inhibit proliferation,Inhibit migration,Inhibit invasion	LINC00485/miR-581/EDEM1	(135)
LINC00543	tissue	↑	Promote EMT,Promote migration	LINC00543/pre-miR-506-3p/FOXQ1	(136)
LINC00586	tissue	↑	Promote migration	LINC00586/LSD1/ASXL1	(137)
LINC00665	cell line	↑	Promote growth,Promote migration,Promote invasion,Inhibit apoptosis	LINC00665/Wnt/β-catenin	(138)
	tissue,cell line	↑	Promote development	LINC00665/miR-138-5p/SIN3A	(139)
LINC00858	tissue	↑	Promote growth,Promote migration	LINC00858/miR-132-3p/IGF2BP1	(140)
LINC00882	tissue,cell line	↑	Promote migration,Promote EMT	LINC00882/miR-3619-5p/TNNB1	(141)
LINC00958	cell line	↑	Promote proliferation,Promote migration, Promote invasion	LINC00958/miR-145-3p/miR-145-3p/CDK1	(142)
	tissue,cell line	↑	Promote proliferation,Inhibit apoptosis, Promote drug resistance,Promote growth	LINC00958/miR-422a/MAPK1	(143)
LINC01021	tissue,cell line	↑	Promote proliferation,Promote colony formation, Promote migration,Inhibit apoptosis	LINC021/IMP2/MSX1/JARID2	(144)
LINC01088	tissue,cell line	↑	Promote proliferation,Promote migration,Promote invasion,Promote immune escape	LINC01088/miR-548b-5p/miR-548c-5p/G3BP1/PD-L1	(145)
LINC01224	tissue,cell line	↑	Promote proliferation,Promote invasion	LINC01224/miR-2467	(146)
	tissue,cell line	↑	Promote proliferation,Promote migration,Promote invasion,Inhibit invasion	YY1/LINC01224/miR-485-5p/MYO6	(147)
LINC01232	cell line	↑	Promote proliferation,Promote angiogenesis, Promote migration,Promote invasion	LINC01232/miR-181a-5p	(148)
LINC01287	tissue	↑	Promote proliferation,Promote migration, Promote invasion,Promote EMT	LINC01287/miR-4500/MAP3K13	(149)
LINC01315	tissue,cell line	↑	Promote migration,Promote invasion, Promote growth	LINC01315/miR-484/DLK1	(150)
	tissue	↑	Promote growth,Promote invasion	LINC01315/miR-205-3p	(151)
	tissue,cell line	↑	Promote migration,Promote growth, Promote EMT	LINC01315/Wnt/β-catenin	(152)
LINC01436	tissue	↑	Promote proliferation	LINC01436/miR-466	(153)
LINC01578	tissue	↑	Enhances metastasis	NF-κB,YY1	(154)
LINC01606	tissue	↑	Promote growth,Promote invasion,Promote stemness	LINC01606/miR-423-5p	(155)
LINC01836	tissue,cell line	↑	Promote proliferation,Promote migration, Promote invasion	LINC01836/miR-1226-3p/SLC17A9	(156)
LINC02038	tissue	↓	Inhibit proliferation,Inhibit vitality,Inhibit migration,Inhibit invasion,Promote apoptosis	M(6)A/LINC02038/miR-552-5p/FAM172A	(157)
LncGMDS-AS1	tissue	↑	Promote proliferation,Promote stemness	GMDS-AS1/HuR-STAT3/Wnt	(61)
MALAT1	tissue,cell line	↑	Promote drug resistance	MALAT1/miR-200s/JNK	(158)
MAPKAPK5-AS1	tissue	↑	Promote proliferation,Promote migration	MK5-AS1/let-7f-1-3p/MK5	(159)
MBNL1-AS1	tissue	↓	Inhibit proliferation	MBNL1-AS1/miR-29c-3p/BVES	(160)
MCF2L-AS	cell line	↑	Promote drug resistance	MCF2L-AS1/miR-105/IL-1β	(161)
MCM3AP-AS1	tissue	↓	Inhibit proliferation,Inhibit migration	MCM3AP-AS1/miR-19a-3p/FOXF2	(162)
MIR155HG	tissue	↑	Promote proliferation,Promote migration,Promote invasion,Promote drug resistance	MIR155HG/miR-650/ANXA2	(163)
MIR497HG	tissue,cell line	↓	Inhibit proliferation,Inhibit migration,Inhibit invasion	MIR497HG/miR-3918/ACTG2	(164)
MNX1-AS1	tissue,cell line	↑	Enhance vitality,Promote invasion,Promote migration	MNX1-AS1/miR-744-5p	(165)
	tissue,cell line	↑	Promote stemness,Promote proliferation, Inhibit invasion,Promote migration	MNX1-AS1/PPFIA4/AKT/HIF-1α	(166)
NEAT1	tissue,cell line	↑	Promote proliferation,Promote invasion	NEAT1/miR-448/ZEB1	(167)
	tissue	↑	Promote proliferation,Promote migration,Promote invasion	NEAT1/miR-216b/YY1	(168)
NEF	cell line	↓	Promote drug resistance	lncRNA-NEF/DOK1/MEK/ERK	(169)
OTUD6B-AS1	cell line	↓	Promote drug resistance	OTUD6B-AS1/HuR/TRIM16/GPX	(170)
	tissue,cell line	↓	Inhibit proliferation,Inhibit migration,Inhibit invasion,Inhibit EMT,Promote apoptosis	OTUD6B-AS1/miR-21-5p/PNRC2	(171)
PCAT1	cell line	↑	Promote proliferation,Promote migration, Promote EMT	PCAT1/miR-329-3p/Netrin-1	(172)
PCGEM1	tissue	↑	Promote proliferation,Promote invasion, Promote migration	PCGEM1/miR-129-5p/SOX4	(173)
	tissue,cell line	↑	Promote proliferation,Promote migration, Inhibit apoptosis	PCGEM1/miR-433-3p/CTCF	(174)
PROX1	tissue,cell line	↑	Promote proliferation,Promote migration, Promote invasion,Promote immune escape	PROX1-AS1/miR-520d/PD-L1	(175)
PROX1-AS1	tissue,cell line	↑	Promote proliferation,Promote migration,Promote invasion	PROX1-AS1/miR-326/FBXL20	(176)
PSMB8-AS1	cell line	↑	Promote proliferation,Promote migration,Promote invasion,Promote EMT,Inhibit apoptosis	PSMB8-AS1/miR-1299/PSMB8-AS1	(177)
LBX2-AS1	tissue,cell line	↑	Promote proliferation,Promote migration,Promote invasion	LBX2-AS1/PTBP1	(178)
PVT1	cell line	↑	Promote proliferation,Promote migration	PVT1/miR-24-3p/NRP1	(179)
	tissue	↑	Promote proliferation,Promote migration	PVT1/miR-1207-5p/Wnt6/β-catenin2	(180)
	tissue	↑	Promote proliferation,Inhibit apoptosis	PVT1/miR-761/MAPK1	(181)
	tissue	↑	Promotes proliferation, migration, invasion	PVT/miR-30d-5p/RUNX2	(182)
RAD51-AS1	tissue,cell line	↓	Promote proliferation,Promote migration, Promote invasion	RAD51-AS1/miR-29b/c-3p/NDRG2	(183)
SNHG11	tissue,cell line	↑	Promote drug resistance,Promote proliferation,Promote migration, Inhibit apoptosis	SNHG11/miR-1207-5p/ABCC1	(184)
SNHG15	tissue	↑	Promote proliferation,Promote drug resistance	TYMS,BCL2,GLUT1,PKM2	(185)
	cell line	↑	Promote proliferation,migration	Slug	(186)
SNHG16	cell line	↑	Promote proliferation,Promote migration,Promote invasion,Promote EMT	SNHG16/miR-124-3p/MCP-1	(187)
	tissue,cell line	↑	Promote proliferation	SNHG16/miR-214-3p/ABCB1	(188)
	cell line	↑	Promote colony formation,Promote proliferation,Promote migration,Promote invasion,Promote EMT	SNHG16/YAP1/TEAD1	(189)
SNHG17	cell line	↑	Elevates proliferation, migration, invasion, Promote colony formation	SNHG17/miR-375/CBX3	(190)
SNHG8	cell line	↑	Promote growth,Promote proliferation, Promote EMT	SNHG8/AKT/AMPK/mTOR	(191)
SOX2OT	tissue	↑	Promote proliferation,Promote migration, Promote invasion,Promote xenotransplantation	SOX2OT/miR-194-5p/SOX5	(192)
SPINT1-AS1	cell line	↑	Promote proliferation,Promote migration, Inhibit apoptosis	SPINT1-AS1/miR-214/HDGF	(193)
	cell line	↑	Promote proliferation,Promote migration, Promote invasion	SPINT1-AS1/miR-433-3p/E2F3	(194)
SURC	tissue	↑	Promote proliferation,Promote colony formation, Promote cell cycle,Promote growth	SURC/miR-185-5p/CCND2	(195)
TDRG1	cell line	↑	Promote stemness	TDRG1/miR-873-5p/PRKAR2	(196)
TUG1	tissue	↑	Promote proliferation,Promote migration	TUG1/miR-195-5p/HDGF/DDX5/β-catenin	(197)
	tissue,cell line	↑	Promote proliferation,Promote migration,Promote invasion,Inhibit invasion	TUG1/miR-542-3p/TRIB2	(198)
	cell line	↑	Promotes proliferation, migration, invasion, EMT, inhibits apoptosis	TUG1/miR-26a-5p/MMP14/p38 MAPK/Hsp27	(199)
UBXN10-AS1 SLIT3	tissue	↓	Promote proliferation,Promote migration	UBXN10-AS1/miR-515-5p/SLIT3	(200)
UCA1	cell line	↑	Inhibit invasion,Promote drug resistance	UCA1/miR-495-HGF/c-MET	(201)
	tissue,cell line	↑	Promote proliferation,Promote migration, Promote invasion	UCA1-miR-495-SP1/SP3	(202)
USP30-AS1	tissue	↓	Inhibit development	USP30-AS1/miR-765	(203)
XIST	tissue,cell line	↑	Promote growth	XIST/GRHL2/miR-448	(204)
	tissue,cell line	↑	Promote proliferation,Promote migration, Promote invasion	XIST/microRNA-338-3p/PAX5	(205)
	tissue,cell line	↑	Promotes proliferation	XIST/miR-34a/Wnt/β-catenin	(206)
XLOC_006390	tissue,cell line	↑	Inhibit apoptosis,Promote migration,Promote invasion	lncRNA-XLOC/u 006390/miR-296/ONECUT2	(207)
ZFAS1	tissue,cell line,plasma	↑	Increases proliferation,invasion, EMT, inhibits apoptosis,regulates cellcycle	ZEB1,E-cadherin,ZO-1,vimentin,N-cadherin	(208)
	cell line	↑	Promote migration	SOX4/miR-34b/ZFAS1	(209)

"↑" indicates that the expression of lncRNA in colorectal cancer is increased, and "↓" indicates that the expression of lncRNA in colorectal cancer is decreased. "/" refers to the part not mentioned in the article.

## Regulation of cell proliferation by LncRNAs

Increasing research evidence suggests that lncRNAs play a crucial role in regulating cellular proliferation. Many studies have shown that abnormal expression of lncRNAs is closely associated with the regulation of proliferation in CRC cells. LncRNAs can promote and inhibit cancer cell proliferation. Generally speaking, lncRNAs that promote cancer cell proliferation are highly expressed in cancer, while lncRNAs that inhibit cancer cell proliferation are low expressed in cancer.

Studies on lncRNAs that promote CRC proliferation are as follows. Guo et al. found that AGAP2-AS1 can be transcriptionally activated by E2F transcription factor 4(E2F4). Downregulating AGAP2-AS1 impairs the proliferation of CRC cells. Mechanistically, AGAP2-AS1 enhances CFL1 expression by competitively binding with miR-182-5p in CRC, providing a possible target for therapeutic intervention (70). When AK093407 is silenced, the proliferation of HCT-15 and HCT-116 cells is reduced (72). ASB16-AS1 promotes the proliferation of CRC cells through the ASB16-AS1-miR-185-5p/TEAD1 axis (73). BBOX1-AS1 promotes the progression of CRC by acting as a sponge for hsa-miR-361-3p and upregulating SH2B1 (75). Silencing CASC9 can not only downregulate AKT3 expression by reducing the competitive binding of CASC9 with miR-576-5p, thereby inhibiting CRC cell proliferation (210), it can also inhibit the proliferation of CRC cells through the miR-542-3p/integrin-linked kinase pathway (80), CCAT2 is highly overexpressed in microsatellite stable CRC and can enhance tumor growth by activating the WNT signaling pathway through the CCAT2/miR-17-5p/TCF7L2 axis (86). Studies have found that by overexpressing or knocking down CCAT2, reducing CCAT2 can increase miR-145, thereby inhibiting the proliferation of cancer cells (211). A study indicated that CCDC144NL-AS1 is upregulated in CRC tissues and cells, and the elevated expression of CCDC144NL-AS1 promotes cell proliferation through the miR-363-3p/GALNT7 axis (212). Another study established cell models with either the gain or loss of function of DANCR. It was discovered that DANCR promotes CRC cell proliferation through the miR-185-5p/HMGA2 axis (92). Research utilizing public datasets and experimental evaluation has explored the function and expression of DICER1-AS1 in CRC. It was found that the upregulation of DICER1-AS1 promotes CRC proliferation *in vitro* by acting as a sponge for miR-650, thereby activating the MAPK/ERK signaling pathway (92). Further research has revealed the DLGAP1-AS2/CTCF/Myc axis as an oncogenic regulatory pathway in CRC, promoting CRC cell proliferation (213). Multiple studies have shown that ELFN1-AS1 is highly expressed in CRC and promotes CRC proliferation through various regulatory axes (95, 214, 215). Overexpression of FAM230B promotes tumor growth by increasing the levels of immature miR-1182 in CRC cells, while simultaneously inhibiting the expression levels of mature miR-1182 (101). Another study used lentiviral transfection to knock down FEZF1-AS1 in CRC cells and found that FEZF1-AS1 likely promotes CRC proliferation through the miR-92b-3p/ZIC5 axis by activating the PI3K/AKT signaling pathway (105). Further research has discovered that knocking out FEZF1-AS1 may inhibit cell proliferation through the miR-632/FAM83A axis (106). FOXP4-AS1 acts as a molecular sponge for miR-423-5p, with NACC1 being a direct target of miR-423-5p.Overexpression of FOXP4-AS1 promotes the proliferation of HCT116 cells (108). GAS6-AS1 acts as an oncogene by competitively binding with miR-370-3p and miR-1296-5p. This interaction leads to the upregulation of TRIM14, which positively regulates CRC proliferation *in vitro* (111). GATA2-AS1 is highly expressed in CRC cells, and knocking down GATA2-AS1 impedes the proliferation of CRC cells (216). Guo et al. found that HCG11 enhances cell proliferation by targeting the miR-26b-5p/ARPP19 axis (116). Another found that silencing HCG11 inhibits the proliferation of CRC cells through the miR-144-3p/PDK4 pathway (117). HOTAIR regulates the levels of HNF4α by recruiting SNAIL. Knocking down HOTAIR inhibits the proliferation of CRC cell lines *in vitro* (124). Deng and colleagues discovered that knocking out HOXB-AS4 inhibits proliferation, and functional restoration experiments further confirmed the critical role of the HOXB-AS4/miR-140-5p/HDAC7 axis in regulating the malignant phenotype of CRC cells (125). Liu and colleagues found that silencing the lncRNA IGFL2-AS1 impedes the malignant proliferation of HCT116 cells and facilitates miR-433-3p-mediated inhibition of PAK4 transcription. Conversely, overexpression of lncRNA IGFL2-AS1 has the opposite effect, promoting cell proliferation by inhibiting miR-433-3p activity and thereby enhancing PAK4 transcription (127). Another study on IGFL2-AS1 also observed that it is highly upregulated in CRC tumor tissues and cells, functionally promoting CRC cell proliferation *in vitro*. Mechanistic investigations revealed that IGFL2-AS1 elevates CA9 levels by affecting the degradation pathway of HIF-1α (128). Overexpression of LBX2-AS1 promotes the proliferation of CRC cells through the miR-627-5p/RAC1/PI3K/AKT pathway (130). *In vitro*, LEF1-AS1 mediates the proliferation of CRC cells through the LEF1-AS1/LEF1/FUT8 axis (131). Liang et al. demonstrated that both loss and gain of function of LINC00174 promote CRC cell proliferation *in vitro*. Mechanistic experiments revealed that LINC00174 binds to miR-2467-3p, enhancing the expression and function of USP21, which in turn affects the proliferation of CRC cells (132). RNA immunoprecipitation (RIP) and RNA pull-down experiments have confirmed that LINC00883 binds with miR-577, and miR-577 interacts with FKBP14. Disrupting the expression of LINC00883 inhibits the proliferation of CRC cells via the miR-577/FKBP14 axis (217). Deficiency of LINC00958 inhibits the proliferation of cancer cells. A study found that LINC00958 functions by interacting with miR-145-3p and modulating the miR-145-3p/CDK1 axis, thereby influencing the proliferation dynamics of the cancer cells (142), Another study found that the pro-proliferative function of LINC00958 is facilitated through the miR-422a/MAPK1 pathway. This interaction contributes to the regulatory mechanism by which LINC00958 enhances cell proliferation (143). Wu et al. confirmed that LINC021 is significantly upregulated in CRC cell lines and clinical tissues, and showed that the oncogenic LINC021 specifically binds with the m(6)A “reader” IMP2 protein, enhancing the mRNA stability of MSX1 and JARID2 through m(6)A regulatory mechanisms during CRC tumorigenesis and pathogenesis (144). Li’s findings suggest that LINC01088 directly targets miR-548b-5p and miR-548c-5p, enhancing the expression of G3BP1 and PD-L1, and thereby promoting the proliferation of CRC cells (145). Silencing LINC01224 inhibits the proliferation of CRC cells. This effect can be reversed by co-transfecting with an miR-2467 inhibitor, indicating that miR-2467 negatively regulates LINC01224. RNA-binding protein immunoprecipitation (RIP) assays further confirm that the inhibition of LINC01224 by miR-2467 is dependent on the RNA-induced silencing complex (RISC) (146) In CRC tissue samples and cell lines, the abundance of LINC01224 is elevated. Knocking down LINC01224 inhibits CRC cell proliferation. Mechanistic studies have shown that YY1-induced LINC01224 regulates CRC proliferation through the miR-485-5p/MYO6 axis (147). It has been observed that LINC1436 can reduce the level of mature miR-466 without affecting its precursors. Proliferation studies indicated that the overexpression of LINC01436 counteracted the reduction in cell proliferation caused by miR-466. Thus, LINC01436, which is found at elevated levels in CRC, enhances cancer cell growth by inhibiting the maturation of miR-466 (153). Ye et al. found that knocking down GMDS-AS1 impairs CRC cell proliferation both *in vitro* and *in vivo*. Through RNA sequencing (RNA-seq) and mass spectrometry (MS) studies, it was discovered that in CRC cells, GMDS-AS1 physically interacts with the RNA-binding protein HuR. This interaction protects HuR from polyubiquitination and proteasomal degradation, highlighting a crucial mechanism by which GMDS-AS1 contributes to the stabilization of proteins involved in cancer cell proliferation (61). The lncRNA MNX1-AS1/PPFIA4 activates the downstream AKT/HIF-1α signaling pathway to promote COAD proliferation (166). NEAT1 can regulate the miR-448/ZEB1 axis (167), and also directly acts as a molecular sponge for miR-216b, which in turn activates YY1 to promote CRC proliferation (168). PCGEM1 is elevated in CRC tissues and cells, and it regulates CRC proliferation through the miR-129-5p/SOX4 axis and the miR-433-3p/CTCF axis (173, 174). PVT1 promotes CRC proliferation through a variety of regulatory pathways, these pathways include the PVT1/miR-24-3p/NRP1 axis, PVT1/miR-1207-5p/Wnt6/β-catenin axis,PVT1/miR-761/MAPK1 axis and PVT1/miR-30d-5p/RUNX2 axis (179–182). Huang et al. found that SNHG11 is overexpressed in bevacizumab-resistant CRC tissues and cells. Knocking down SNHG11 inhibits cell proliferation, possibly through the miR-1207-5p/ABCC1 axis (184). SNHG15 promotes CRC cell proliferation, while knocking down SNHG15 inhibits it. RT-qPCR and Western blot analyses confirm that SNHG15 enhances the expression of TYMS, BCL2, GLUT1, and PKM2 in CRC cells (185). Additionally, another study suggests that SNHG15 interacts with the ubiquitin-proteasome system to block the degradation of Slug, thereby inhibiting its breakdown (186). SNHG16 regulates CRC proliferation through multiple pathways, including SNHG16/miR-124-3p/MCP-1 axis, SNHG16/miR-214-3p/ABCB1 axis and SNHG16/YAP1/TEAD1 axis (187–189). Functionally, SNHG17 can promote the proliferation of COAD cells *in vitro*, and its effect is mediated through the miR-375/CBX3 axis (190). Islam et al. used dicer-substrate siRNA transfection to modulate the expression of SNHG8 in the HCT-116 and SW480 cell lines. The knockdown of SNHG8 induced autophagy and apoptosis pathways through the AKT/AMPK/mTOR axis, significantly reducing the proliferation of CRC cells (191). SOX2OT is an oncogene in human CRC tissues and cell lines. Silencing of SOX2OT *in vitro* inhibits cell proliferation in CRC cells. Mechanistically, SOX2OT acts as a ceRNA that upregulates SOX5 by sponging miR-194-5p (192). SPINT1-AS1 is upregulated in CRC, and the silencing of SPINT1-AS1 inhibits the proliferation of CRC cells. SPINT1-AS1 mediates the expression of HDGF by targeting miR-214 (193). Another study indicated that knocking down SPINT1-AS1 weakened the proliferation of KRAS-silenced CRC cells. Rescue experiments confirmed the regulatory effect of the SPINT1-AS1/miR-433-3p/E2F3 axis on the proliferation of CRC cells (194). Li et al. found that mutated APC genes can promote the transcription of SURC in CRC by reducing the degradation of β-catenin. Functional assays showed that knockdown of SURC inhibits the proliferation of CRC cells, potentially through the SURC/miR-185-5p/CCND2 axis (195). TUG1 is involved in the regulation of proliferation in CRC through multiple pathways. Xia et al. reported that TUG1, stabilized by IGF2BP2, promotes CRC cell proliferation through the miR-195-5p/HDGF/DDX5/β-catenin axis (197). Liu et al. found that knockdown of TUG1 inhibits tumor growth *in vivo*, where TUG1 interacts with miR-542-3p, affecting the expression of TRIB2 and inhibiting CRC proliferation through the Wnt/β-catenin pathway (198). Tian et al. reported that TUG1 promotes CRC proliferation both *in vivo* and *in vitro* by regulating the miR-26a-5p/MMP14/p38 MAPK/Hsp27 axis (199). Liu et al. transfected pcDNA-UCA1 plasmids to overexpress UCA1 and found that dysregulation of the UCA1/miR-495/SP1/SP3 axis in CRC leads to malignant proliferation of CRC cells (202). XIST is involved in the regulation of CRC proliferation through various pathways. Yan et al. reported that XIST is highly expressed in CRC tissues and cells, and its downregulation *in vitro* inhibits CRC cell proliferation, which is regulated through the XIST/miR-448/GRHL2 axis (204), Li et al. found that knockdown of XIST inhibits cell proliferation through the miR-338-3p/PAX5 axis (205). Sun et al.’s study indicates that XIST regulates cell proliferation through the miR-34a/Wnt/β-catenin signaling pathway (206). In cells where ZFAS1 is knocked down, an increase in the expression of epithelial markers E-cadherin and ZO-1 is observed, along with a decrease in the expression of mesenchymal markers vimentin and N-cadherin (208).

The research on lncRNAs that inhibit the proliferation of CRC is as follows. CASC2 inhibits the expression of miR-18a-5p by acting as a molecular sponge for miR-18a-5p, positively regulates the expression of BTG3, and thereby achieves the suppression of CRC cell proliferation (78). Experimental studies both *in vitro* and *in vivo* have confirmed that upregulating CYP1B1-AS1 markedly inhibits CRC cell proliferation. Additionally, CYP1B1-AS1 directly binds to and negatively regulates NOP58. The effects of CYP1B1-AS1 can be reversed by the overexpression of NOP58 (90). Xiong et al. transfected HCT-116 cells with si-GAS5 to inhibit GAS5 expression and found that this suppression led to an upregulation of miR-21. This increase in miR-21 subsequently affected the PTEN/Akt signaling pathway, promoting cell proliferation (110). Yi et al.found that overexpression of HAND2-AS1 in CRC cell lines using an expression vector, revealed that HAND2-AS1 suppresses CRC cell proliferation by acting as a molecular sponge for miR-3118, which affects the LEPR axis (115). Li et al. found that knocking down LINC00485 in FHC cells promoted cell proliferation. Mechanistic studies revealed that LINC00485 inhibits CRC tumor proliferation by targeting the miR-581/EDEM1 axis (135). In CRC tissues, overexpressing LINC02038 significantly inhibits CRC cell proliferation. It acts as a sponge for miR-552-5p, protecting its target gene, FAM172A, from degradation (157). MBNL1-AS1 primarily inhibits the proliferation of CRC cells by regulating the miR-29c-3p/BVES signaling pathway (160). OTUD6B-AS1 is expressed at lower levels in CRC tissues and cells. Mechanistic studies have shown that OTUD6B-AS1 inhibits CRC proliferation by sponging miR-21-5p and upregulating PNRC2 (171). MBNL1-AS1 inhibits CRC cell proliferation by regulating the miR-29c-3p/BVES signaling pathway (218). Zhang et al. observed that RPL34-AS1 may act as a molecular sponge for miR-3656, inhibiting CRC cell proliferation (219).

## LncRNAs regulate cell apoptosis

One of the important reasons for the infinite proliferation of cancer cells is that their apoptosis function is inhibited, thus allowing cells to escape programmed death and continue to proliferate, and lncRNA has the function of regulating apoptosis of cancer cells (220, 221). The following is a summary of research on lncRNA that promotes cell apoptosis.

In the study by Xue et al., it was found that silencing AK093407 increased the apoptosis rate of HCT-15 and HCT-116 cells (72). Yu et al. reported that silencing ASB16-AS1 accelerates apoptosis in CRC cells by regulating the miR-185-5p/TEAD1 axis (73). Another study indicated that knocking down BBOX1-AS1 reduces apoptosis, which may be achieved by upregulating SH2B1 through the sponge effect on hsa-miR-361-3p (75). The study by Li et al. reported that knocking down BCAR4 promotes apoptosis in CRC cells, which can be reversed by overexpressing RAB5C.The related regulatory axis is the BCAR4/miR-483-3p/RAB5C axis (76). Liu’s report indicates that silencing CASC9 can promote apoptosis by downregulating AKT3 expression through reducing the competitive binding of CASC9 with miR-576-5p (222). Liang et al. found that Duxap8 inhibits apoptosis *in vitro*. Mechanistically, Duxap8 is primarily located in the cytoplasm and acts as a competitive endogenous RNA, affecting apoptosis by upregulating ZNF277 through the sponge effect on miR-519b-3p (94). FOXP4-AS1 was found to act as a molecular sponge for miR-423-5p in HCT116 cells and animal models, with NACC1 being a direct target of miR-423-5p.Overexpression of FOXP4-AS1 inhibits apoptosis in CRC cells (108). Guo et al.found that knocking down HCG11 promoted apoptosis. Through bioinformatics analysis and mechanistic studies, HCG11, primarily located in the cytoplasm, has been shown to competitively bind with miR-26b-5p to regulate the expression of cAMP-regulated phosphoprotein 19 (ARPP19) (116). Deng et al. discovered that knocking out HOXB-AS4 can promote apoptosis.HOXB-AS4 acts as a molecular sponge by binding and adsorbing miR-140-5p to regulate the expression of histone deacetylase 7 (HDAC7) (125). Liang et al. demonstrated through loss and gain of function studies of LINC00174 that it enhances resistance to apoptosis in CRC cells *in vitro* via the miR-2467-3p/USP21 axis (132). Another study reported that LINC00665 stimulates apoptosis in CRC by activating the Wnt/β-catenin signaling pathway (138). LINC00958 promotes the expression of MAPK1 by targeting miR-422a and inhibits apoptosis (143). Wu et al. found that LINC021 directly recognizes the IMP2 protein, which enhances the mRNA stability of transcripts such as MSX1 and JARID2 by recognizing their m(6)A-modified element RGGAC, thereby reducing apoptosis (144). Xu et al. found that knocking down PCGEM1 inhibits apoptosis in CRC cells (174). Liu et al. believe that knocking down the expression of PVT1 promotes apoptosis in CRC cells through the miR-761/MAPK1 axis (181). Huang et al. found that knocking down SNHG11 promotes apoptosis in bevacizumab-resistant CRC cells, potentially through the miR-1207-5p/ABCC1 axis (184). The silencing of SPINT1-AS1 mediates the expression of HDGF by targeting miR-214, increasing apoptosis in CRC cells (193). Ma et al.’s study reveals that silencing XLOC_006390 inhibits apoptosis in CRC through the regulation of the miR-296/ONECUT2 axis (207). Fang et al. believe that ZFAS1 prevents apoptosis. Knocking down ZFAS1 can reduce the expression of ZEB1 and increase epithelial markers E-cadherin and ZO-1, while decreasing mesenchymal markers vimentin and N-cadherin (208).

The following is a summary of relevant studies on lncRNAs that inhibit cell apoptosis.

A study indicates that GAS5 is significantly reduced in CRC tumor tissues and cells, and knocking down GAS5 can promote apoptosis in cancer cells (109). Wang et al.’s study suggests that LINC00261 may promote apoptosis by downregulating nuclear β-catenin through inhibiting the translocation of β-catenin from the cytoplasm to the nucleus or by promoting the degradation of β-catenin and inhibiting the activation of the Wnt pathway (133). Research has found that the m(6)A/LINC02038/miR-552-5p/FAM172A axis may represent a novel anti-tumor pathway. Overexpression of LINC02038 can accelerate apoptosis in CRC cells through this axis (157). Cai et al. suggest that the overexpression of OTUD6B-AS1 promotes apoptosis through the OTUD6B-AS1/miR-21-5p/PNRC2 axis (171).

## LncRNAs regulate invasion and metastasis

Invasion refers to the ability of cancer cells to penetrate from the original tumor site into surrounding normal tissues, while metastasis is the process by which cancer cells spread through the blood or lymphatic system to form new tumors at other sites in the body. The invasion and metastasis of CRC cells are related to a variety of factors, including cellular pathways and molecular mechanisms, epithelial-mesenchymal transition (EMT), and the influence of microorganisms, all of which together promote the aggressive and metastatic characteristics of CRC (223, 224). LncRNAs can also promote and inhibit the migration and invasion of cancer cells. The following is a brief summary of the research on LncRNAs promoting cancer migration and invasion.

The expression of AGAP2-AS1 is significantly elevated in CRC cells. Downregulation of AGAP2-AS1 can attenuate the migration and invasion of CRC cells. Mechanistically, miR-182-5p is a downstream target molecule of AGAP2-AS1, and CFL1 is a target of miR-182-5p (70). Yu et al. found that ASB16-AS1 is highly expressed in CRC cells. Silencing ASB16-AS1 suppressed the proliferation, migration, and invasion of CRC cells. Mechanistic experiments revealed that ASB16-AS1 drives the progression of CRC by regulating the miR-185-5p/TEAD1 axis (73). Yue et al. found that the levels of lncRNA ATB are higher in three highly invasive CRC cell lines compared to three low-invasive cell lines (74). Knockdown of BBOX1-AS1 enhances cancer cell migration and invasion, with its regulatory axis being hsa-miR-361-3p/SH2B1 (75). BVES-AS1 can encode a micropeptide that promotes the migration and invasion of CRC cells *in vitro (* 77). Zhang et al. discovered that the CASC9/miR-542-3p/integrin-linked kinase regulatory axis is involved in the invasion and migration of CRC cells (80). The upregulation of CCAT1 expression promotes cell proliferation and invasion (83). Depletion of CERS6-AS1 inhibits cell migration and invasion through the miR-15b-5p/SPTBN2 axis (88). Knockdown of CYTOR reduces the migration and invasion of colon cancer cells (91). The DANCR/miR-185-5p/HMGA2 axis is involved in the migration and invasion processes of CRC (92). The upregulation of DICER1-AS1 promotes CRC migration and invasion by activating the MAPK/ERK signaling pathway through a sponge-like effect on miR-650 (93). Bin et al. found that EPB41L4A-AS1 promotes the invasion and migration of CRC (97). FEZF1-AS1 can activate the PI3K/AKT signaling pathway to promote the proliferation and invasion of RKO cells (105), it can also promote CRC migration and invasion through the miR-632/FAM83A axis (106). ATF3-activated FOXP4-AS1 enhances CRC migration and invasion by regulating the miR-423-5p/NACC1 axis (108). GAS6-AS1 promotes TRIM14-mediated CRC cell migration and invasion through a ceRNA network and FUS-dependent mechanism (111). Guo et al. reported that upregulated HCG11 in CRC cells can promote cell migration and invasion by targeting the miR-26b-5p/ARPP19 axis (116), Cui reported another regulatory axis, HCG11/miR-144-3p/PDK4 (117). Upregulated HNF1A-AS1 promotes migration and invasion of colon cancer cells *in vitro* and *in vivo (* 118). IGFL2-AS1 promotes CRC cell migration and invasion *in vitro* and accelerates the occurrence of CRC *in vivo (* 128). Fang’s research team reported that overexpression of LBX2-AS1 significantly promotes the metastasis of colon cancer cells (130). Liang and others designed loss and gain of function experiments for LINC00174, proving its key role in promoting CRC cell migration and invasion *in vitro (* 132). LINC00473 acts as a ceRNA for miR-195 to promote cell invasion in CRC (134). The expression of LINC00473 accelerates the aggressiveness of CRC (134). Knockdown of LINC00485 promotes migration and invasion in FHC cells, while overexpression weakens these abilities in LoVo cells (135). LINC00665, acting as a sponge for miR-214-3p, upregulates CTNNB1 expression, thereby activating the Wnt/β-catenin signaling pathway and regulating CRC cell migration and invasion (138). LINC00958 directly binds to miR-145-3p, which interacts with downstream effector CDK1, forming the LINC00958 regulatory axis for CRC migration and invasion (142). Knockdown of LINC01088 inhibits proliferation, migration, invasion, and immune evasion of CRC cells. Mechanistically, LINC01088 directly binds to miR-548b-5p and miR-548c-5p, with miR-548c-5p significantly upregulating the expression of Ras GTPase activating protein binding protein 1 (G3BP1) and programmed death ligand 1 (PD-L1), thereby regulating CRC cell migration and invasion (145). LINC01224 can promote CRC invasion through a sponge-like mechanism on miR-2467 (146), and can also regulate migration and invasion through the miR-485-5p/MYO6 axis (147). Knockdown of LINC01287 inhibits colon cancer cell migration and invasion through the LINC01287/miR-4500/MAP3K13 axis (149). Li et al. reported that silencing of LINC01315 regulates CRC cell migration and invasion through the miR-484/DLK1 axis (150), while Liang and others reported another regulatory axis: LINC01485/miR-383-5p/KRT80 (151). Liu’s team used gain and loss of function assays to show that LINC01578 enhances colon cancer cell vitality and migration rate *in vitro*, and enhances colon cancer liver metastasis *in vivo* (154). LINC01606 acts as an oncogene promoting colon cancer cell invasion *in vitro* and *in vivo*, and is associated with the Wnt/β-catenin signaling pathway (155). Zhou et al. found that knockdown of MIR155HG inhibits CRC cell migration and invasion, regulated through the miR-650/ANXA2 axis (163). Tang et al.’s study suggests that MIR497HG, as a ceRNA regulating the miR-3918/ACTG2 axis, plays a key role in CRC cell migration and invasion (218). MNX1-AS1 regulates miR-744-5p (165) or the PPFIA4/AKT/HIF-1α axis (166), playing a critical role in the invasion and migration of CRC cells.NEAT1 promotes the expression of ZEB1 by targeting miR-448 (167), or enhances the proliferation and invasion of CRC through the miR-216b/YY1 axis (168). Guan et al. found that PCGEM1 mediates the invasion and migration of CRC cells through targeting the miR-129-5p/SOX4 axis (173). Li et al.’s research confirms that PROX1-AS1 can absorb miR-520d to upregulate PD-L1 in CRC (175), while Liu et al. discovered that the transcription factor SP1 activates the PROX1-AS1/miR-32/FBXL20 axis (176), both of which are involved in the regulation of CRC cell migration and invasion. Yu et al.’s results indicate that PVT1 can promote the metastasis of colon cancer by inhibiting the miR-30d-5p/RUNX2 axis (182). Chen et al. found that SNHG16 facilitates CRC cell migration through the miR-124-3p/MCP-1 axis (187). Xiang et al. discovered that SNHG16 acts as a miRNA sponge to sequester miR-195-5p on Ago2, thereby protecting YAP1 from inhibition and subsequently promoting CRC cell migration and invasion (189). Liu et al. demonstrated a new triad involving SNHG17/miR-375/CBX3 that participates in the migration and invasion of COAD (190). SOX2OT,acting as a ceRNA, upregulates SOX5 through a sponge-like effect on miR-194-5p, regulating CRC cell migration and invasion (192). Sui et al. observed that knockdown of SPINT1-AS1 weakens the migration and invasion of CRC cells with silenced KRAS (194). Knockdown of TUG1 not only inhibits CRC migration and invasion through the miR-542-3p/TRIB2 axis (198) but also, by overexpressing it, regulates the miR-26a-5p/MMP14/p38 MAPK/Hsp27 axis *in vitro* and *in vivo* to accelerate cancer invasion (199). Liu et al. found that UCA1 is significantly upregulated in CRC tissues. UCA1 enhances the migration and invasion of CRC cell lines (202). Li et al. reported that downregulation of XIST inhibits migration and invasion in CRC cells by regulating the miR-338-3p/PAX5 axis (205). The silencing of XLOC006390 significantly inhibits their viability by inducing apoptosis and limiting the migration and invasion of cancer cells (207). Fang et al.’s study shows that ZFAS1 promotes CRC invasion, and its mechanism is associated with E-cadherin, ZO-1, vimentin, and N-cadherin (208).

The following is an overview of research on LncRNAs inhibiting cancer migration and invasion. Liu et al. found that AK077216 is downregulated in CRA tissues. AK077216 may inhibit the migration and invasion of CRA cells by downregulating miR-34a (71). Overexpression of CASC2 inhibits the migration and invasion capabilities of CRC cells through the miR-18a-5p/BTG3 axis (78). CBR3-AS1 is downregulated in CRC and inhibits miR-29a-mediated cell migration and invasion through a sponge-like mechanism on miR-29a (81). Knockdown of CCL14-AS enhances the invasiveness and lymph node metastatic capability of CRC cells in nude mice, with the CCL14-AS/MEP1A axis playing a key role (87). CYP1B1-AS1 is significantly downregulated in CRC. Experiments conducted *in vitro* and *in vivo* have confirmed that upregulation of CYP1B1-AS1 significantly inhibits the migration and invasion of CRC cells (90). Knockdown of both FER1L4 and p73 enhances the migration and invasion of CRC cells. Mechanistic studies have found that FER1L4 promotes the expression of miR-1273g-3p, which in turn increases the expression of PTEN (103). Another study found that restoration of FER1L4 reduces the expression of miR-106a-5p and significantly affects the migration and invasion of colon cancer cells (104). GAS5 can act as a competitive endogenous RNA for miR-21. The downregulation of GAS5 can promote CRC migration and invasion by activating the miR-21/PTEN/Akt signaling pathway (110). Overexpression of HOXC-AS3 reduces cell migration and invasion (126). Wang et al. reported that LINC00261 is downregulated in colon cancer cell lines, tissues, and cisplatin-resistant cells; overexpression of LINC00261 may inhibit cell migration and invasion (133). Knockdown of LINC00485 promotes migration and invasion in FHC cells, and the miR-581/EDEM1 axis is involved (135). Liu et al. reported that m(6)A/LINC02038/miR-552-5p/FAM172A might be a novel anti-tumor axis, closely related to regulating CRC migration and invasion (157). Cai et al. reported that overexpression of OTUD6B-AS1 inhibits the migration and invasion of CRC cells (171).

## LncRNAs regulate cell cycle

LncRNAs play a crucial role in the development of CRC by regulating the cell cycle. LncRNAs can intervene in the control of the cell cycle through various mechanisms. Research has found that the levels of AK093407 are higher in the CRC cell lines HCT-15 and HCT-116 compared to normal colonic epithelial NM460 cells. When AK093407 is silenced, the cell cycle of HCT-15 and HCT-116 cells stalls at the G1/S phase (72).Overexpression of DANCR promotes the progression of the CRC cell cycle, with the DANCR/miR-185-5p/HMGA2 axis involved in the regulation (92). Interestingly, during the amplification of the full-length cDNA of EVADR (named EVADR-v1), a new/shorter variant (EVADR-v2) was discovered. When both variants, EVADR-v1 and EVADR-v2, are overexpressed in SW480/HCT116 cells, there is an increase in PI3K activity and upregulation of WNT signaling, thereby promoting cell cycle progression (98). Xiong et al. reported that inhibiting GAS5 can upregulate the expression levels of miR-21, decrease G1 phase cells, and increase S phase cells (110). Reports also suggest that increased expression of LINC00473 accelerates the cell cycle process. Li et al. demonstrated through functional assays that knockdown of SURC inhibits the CRC cell cycle (195).

## LncRNAs regulate cancer cell stemness

Yu et al. discovered that ASB16-AS1 is highly expressed in CRC cells, and silencing ASB16-AS1 inhibits the stemness of CRC cells. Mechanistic exploration revealed that ASB16-AS1 drives the progression of CRC by regulating the miR-185-5p/TEAD1 axis (73). Zhao used sphere-formation assays to examine cell stemness and found that depletion of CERS6-AS1 inhibits cell stemness (88). Knockdown of FAM201A inhibits cell stemness, and its regulation is achieved through the miR-3163/MACC1 axis (100). hang et al. demonstrated that Sohlh2 is associated with the inhibition of the LncRNA-H19/miR-141/β-catenin signaling pathway, leading to the suppression of colon CSC (cancer stem cell) stemness (114). Huang et al. used an analysis of stemness-related markers to determine cell stemness, finding that HOTAIR promotes the expression of the miR-211-5p target gene FLT-1, thereby regulating the characteristics of cancer stem cells (CSC) in CRC (120). LINC01606 acts as an oncogene and promotes the stemness of colon cancer cells both *in vitro* and *in vivo*. Mechanistically, LINC01606 enhances the expression of SCD1 and regulates the expression of miR-423-5p as a competitive endogenous RNA, subsequently activating the classic Wnt/β-catenin signaling pathway. Additionally, the transcription factor’s binding to IGHM enhancer 3 (TFE3) increases the transcription of LINC01606 after being recruited to the promoter region of LINC01606 (155). Ye et al.’s study indicates that the lncRNA GMDS-AS1 and its direct target HuR constitutively activate the STAT3/Wnt signaling pathway and promote the stemness of CRC tumors (61). Sun et al. reported that PPFIA4 mediates the expression of lncRNA MNX1-AS1 and affects the stemness of COAD cells. The MNX1-AS1/PPFIA4 activates the downstream AKT/HIF-1α signaling pathway to promote the progression of COAD (166). TDRG1 levels are significantly upregulated in 3D non-adherent spheroids derived from parental CRC cells. Further studies indicate that knockdown of TDRG1 inhibits the stemness of CRC cells (196).

## LncRNAs regulate EMT

LncRNAs also play an important role in the EMT process in CRC.E2F4-stimulated AGAP2-AS1 exacerbates the EMT process in CRC by regulating the miR-182-5p/CFL1 axis (70). lncRNA-ATB activated by TGF-β may contribute to the development of colon cancer by inhibiting E-cadherin expression and promoting the EMT process (74). Zhao et al. reported that CERS6-AS1 also participates in regulating the EMT process (88). Yue et al.’s experiments show that ectopic expression of CYTOR induces EMT (91).Research indicates that knockdown of EPB41L4A-AS1 inhibits EMT (97). Chen et al. reported that GAS6-AS1 positively regulates the CRC EMT process *in vitro* through a ceRNA network and FUS-dependent manner (111). Wu et al. consider HOTAIR to be a pleiotropic regulator involved in EMT (122), and Jin et al. demonstrated that HOTAIR regulates HNF4α levels by recruiting SNAIL, thereby regulating the EMT process in CRC cells (124). Li et al. report that LINC00473 regulates EMT progression by modulating miR-195 expression (134). Zheng et al.’s study shows that LINC00543 enhances CRC cell EMT through the pre-miR-506-3p/FOXQ1 axis (136). Research shows that knockdown of LINC00882 impedes the EMT process in CRC cells (141). Fu et al.’s research found that the LINC01287/miR-4500/MAP3K13 axis promotes EMT in colon cancer (149). Studies also suggest that the LINC01315/Wnt/β-catenin signaling pathway provides new insights into regulating CRC EMT (152). Cai et al.’s study found that the OTUD6B-AS1/miR-21-5p/PNRC2 axis is involved in regulating CRC EMT (171). Research finds that SNHG16 promotes CRC cell EMT through the miR-124-3p/MCP-1 axis (187) and SNHG16/YAP1/TEAD1 axis (189). Islam et al. consider SNHG8 as an oncogene in CRC through the EMT pathway (191). Tian et al. believe that TUG1 accelerates cancer EMT by regulating the miR-26a-5p/MMP14/p38 MAPK/Hsp27 axis *in vitro* and *in vivo* (199). Fang et al.’s study indicates that ZFAS1 may act as an oncogene by regulating ZEB1 to induce EMT (208).

## LncRNAs regulate drug resistance

Drug resistance is a complex process and has always been one of the most unfavorable factors in the process of CRC.Zheng et al.’s research, through microarray screening, identified lncRNAs associated with resistance to oxaliplatin. They experimentally confirmed that the AC092894.1/USP3/AR/RASGRP3 signaling axis is a new option for treating oxaliplatin resistance (69). Li et al. reported that BCAR4 promotes oxaliplatin resistance by inhibiting apoptosis through the BCAR4/miR-483-3p/RAB5C axis (76). Liu found that CCAT1 enhances CRC cell resistance to oxaliplatin by transcriptionally activating CCAT1 through B-MYB, increasing SOCS3 promoter methylation to recruit DNMT1, thereby inhibiting SOCS3 expression and enhancing CRC cell resistance to oxaliplatin (82). Another study found that downregulation of CCAT1 effectively reversed the resistance of HCT 116/5-FU and HT-29/5-FU cells to 5-FU chemotherapy (84). Yang et al. reported that silencing CRNDE promotes apoptosis in CRC cells and enhances sensitivity to cisplatin, possibly through regulation of the Warburg effect mediated by the Akt/mTORC1 pathway (89). Li et al. emphasized the potential of targeting ELFN1-AS1 as a therapeutic agent in cell survival and resistance to oxaliplatin (95). Gao et al., through *in vitro* and *in vivo* xenograft experiments, showed that the specific inhibitor erlotinib enhances the anti-tumor toxicity of 5-Fu by targeting the EGFR/FGD5-AS1/miR-330-3p/HK2 pathway (107). Chen et al. found that overexpression of H19 may be one of the mechanisms by which advanced colon cancer develops resistance to treatment with 1,25(OH)2D3 (113). A study revealed the crucial role and molecular mechanism of HCG11-mediated 5-FU resistance in CRC through regulation of the miR-144-3p/PDK4/glucose metabolism pathway (117). Zhang et al. reported that patients carrying allele 10 and having methylation in the KCNQ1OT1 promoter exhibited the greatest reduction in resistance to colon cancer treatment (129). Research has found that LINC00261 reduces cisplatin resistance in colon cancer *in vivo*, and enhances the anti-colon cancer effects of cisplatin by reducing tumor volume and weight (133). LINC00958 promotes the expression of MAPK1 by targeting miR-422a, inhibiting cell radiosensitivity (143). In Wei’s study on the traditional Chinese medicine Zuojin Wan, it was found that MALAT1 promotes resistance to oxaliplatin in CRC by decreasing the expression of miR-200s. Zuojin Wan may reverse chemotherapy resistance by inhibiting the expression of MALAT1 and regulating the miR-200s/JNK pathway (158). Cai et al. reported that knockdown of MCF2L-AS1 can alleviate the chemoresistance of CRC/OXA cells (161). Zhou et al.’s research reported that MIR155HG promotes CRC progression and enhances CRC cell resistance to oxaliplatin by regulating the miR-650/ANXA2 axis and through M2 macrophage polarization (163). Shi et al.’s work found that lncRNA-NEF plays a key role in mediating oxaliplatin chemotherapy resistance in CRC, providing a promising therapeutic strategy for CRC patients resistant to oxaliplatin (169). Zhang et al. reported that overexpression of OTUD6B-AS1 by binding with HuR stabilizes TRIM16 and increases iron accumulation mediated by GPX4, thus weakening CRC’s radioresistance (170). Another study indicated that exosomal SNHG11 is upregulated in bevacizumab-resistant CRC cells, and SNHG11 contributes to the resistance to bevacizumab in CRC, depending on the regulation of miR-1207-5p and ABCC1 (184). Li et al.’s research suggests that SNHG15 promotes 5-FU chemoresistance in CRC by potentially regulating the expression of TYMS, BCL2, GLUT1, and PKM2 (185). Yuan et al.’s research provides the first evidence that the UCA1-miR-495-HGF/c-MET regulatory network is involved in CRC resistance to cetuximab (201).

## Discussion

CRC is one of the major health challenges globally, with a high mortality rate, especially when the disease is diagnosed at a late stage. To improve the success rate of treatments and the survival rates of patients, the development of reliable early detection biomarkers is particularly crucial. In recent years, researchers have begun to focus on the potential role of LncRNAs in CRC, especially as non-invasive molecular biomarkers (225).

LncRNAs have multifaceted functions in CRC, including the regulation of the cell cycle, proliferation, apoptosis, and metastasis. They often function as ceRNAs that affect the expression of specific miRNAs and subsequently the downstream molecules of these miRNAs. Additionally, LncRNAs play an important role in regulating gene expression, including transcriptional regulation, mRNA stability, and translation. Research indicates that LncRNAs might interact with RNA, DNA, and proteins to form complex regulatory networks, thereby affecting the onset and progression of CRC.Given the stability of LncRNAs in blood and their potential in early CRC detection, they are considered a promising tool for non-invasive testing. Furthermore, studies on LncRNAs have also revealed their potential key roles in regulating various pathological processes related to CRC, such as influencing the aggressiveness and metastatic properties of cancer cells through specific regulatory axes.

At present, the main diagnostic methods for CRC include colonoscopy, tissue biopsy, and serum tumor marker detection. Although these methods are widely used, they have some significant limitations. Colonoscopy is an invasive procedure where patients need to prepare their intestines and may experience discomfort or pain during the examination process (226). In addition, although tissue biopsy is the gold standard for diagnosis, the limitations of sampling may lead to missed diagnosis (227). The detection sensitivity and specificity of serum tumor markers such as CEA and CA19-9 are low, and false positive and false negative results are prone to occur, which cannot accurately reflect early carcinogenesis (228). In terms of treatment, surgical resection is the main treatment for early CRC, while chemotherapy and radiotherapy are used as adjuvant treatments for advanced or postoperative conditions. However, these treatment methods also have certain limitations. Although surgery can remove tumors, it is difficult to completely remove early metastases or small lesions (228). Chemotherapy and radiation therapy may cause serious side effects such as nausea, vomiting, and bone marrow suppression, and some patients may develop drug resistance, leading to poor treatment outcomes (229). In addition, although targeted therapy is effective for patients with certain specific gene mutations, its scope of application is limited and the treatment cost is high (227). LncRNA provides a certain possibility to solve the above problems. In the past decade, numerous studies have shown that lncRNA is stable in the bloodstream and has excellent diagnostic potential, making lncRNA a promising candidate for non-invasive diagnostic testing of CRC (43–46). In the treatment of CRC, LncRNA can serve as a novel therapeutic target, affecting tumor growth and metastasis by regulating the expression of oncogenes and tumor suppressor genes. Combining lncRNA with small molecule drugs or gene therapy to develop novel therapies with stronger targeting and fewer side effects may break through the limitations of existing treatments (230, 231). LncRNA also plays an important role in the mechanism of chemotherapy and radiation resistance. By intervening in the function of these lncRNAs, it is expected to reverse drug resistance and improve treatment efficacy (232, 233).

Although lncRNAs have broad application prospects, their application in clinical diagnosis and treatment still faces multiple challenges:1. In the previous chapters, we listed many lncRNAs with a diagnostic AUC value greater than 0.9 in **Table 1**, but most of the experimental and control groups included in these studies were less than 100 cases, so there may be a large bias in the research results. In addition, the research is also limited by age, race, gender and other factors. In order to obtain more credible results, these studies should be verified with a larger sample size and wider population distribution; 2. Currently, there are various methods for detecting lncRNA, including qRT qPCR, RNA seq, Sanger sequencing, and Northern blot. The standardization and optimization of these methods still need further development to ensure consistency and reproducibility in different laboratory and clinical environments (234, 235). 3.The data analysis process of high-throughput lncRNAs is complex and requires high-level professional knowledge and computing resources (236, 237). 4.Although many lncRNAs have been found to be associated with cancer, research on their specific functions and mechanisms is still in its early stages. The current research lacks a comprehensive understanding of the mechanism of action of lncRNA, which limits its potential in clinical applications (238, 239). 5.Treatment strategies based on lncRNA, such as siRNA and antisense oligonucleotides (ASO), face issues such as low delivery efficiency, poor specificity, and off target effects, which further limit their therapeutic efficacy (240).

## Conclusion

In summary, research on LncRNAs provides a new perspective for understanding the molecular mechanisms of CRC and brings hope for the development of new diagnostic and therapeutic strategies. Future studies are needed to further explore the specific functions of these molecules in CRC and their potential clinical applications, with the hope of bringing more effective treatment options and better survival prospects for CRC patients.

## Author contributions

YNL: Conceptualization, Data curation, Formal analysis, Funding acquisition, Investigation, Methodology, Project administration, Resources, Software, Supervision, Validation, Visualization, Writing – original draft, Writing – review & editing. WZ: Investigation, Visualization, Writing – review & editing. ZL: Investigation, Writing – review & editing. HX: Investigation, Methodology, Writing – review & editing. YL: Investigation, Software, Writing – review & editing. ZZ: Conceptualization, Data curation, Methodology, Writing – review & editing.
